# The Role of Personality and Top Management Support in Continuance Intention to Use Electronic Health Record Systems among Nurses

**DOI:** 10.3390/ijerph191711125

**Published:** 2022-09-05

**Authors:** Adi Alsyouf, Awanis Ku Ishak, Abdalwali Lutfi, Fahad Nasser Alhazmi, Manaf Al-Okaily

**Affiliations:** 1 Department of Managing Health Services and Hospitals, Faculty of Business Rabigh, College of Business (COB), King Abdulaziz University, Jeddah 21991, Saudi Arabia; 2 School of Business Management, College of Business, University Utara Malaysia (UUM), Sintok 06010, Kedah Darul Aman, Malaysia; 3 Department of Accounting, College of Business Administration, King Faisal University, Al-Ahsa 31982, Saudi Arabia; 4 Department of Health Services and Hospital Administration, Faculty of Economics and Administration, King Abdulaziz University, Jeddah 21589, Saudi Arabia; 5 School of Business, Jadara University, Irbid 733, Jordan

**Keywords:** continuance intention, UTAUT, five-factor model, electronic health records, nurses, post-adoption

## Abstract

This study examines nurses’ Continuance Intention (CI) to use electronic health records (EHRs) through a combination of three conceptual frameworks: the Unified Theory of Acceptance and Use of Technology (UTAUT), the theory of expectation-confirmation (ECT), and the Five-Factor Model (FFM). A model is developed to examine and predict the determinants of nurses’ CI to use EHRs, including top management support (TMS) and the FFM’s five personality domains. Data were collected from a survey of 497 nurses, which were analyzed using partial least squares. No significant relationship was found between TMS and CI. The study revealed that performance expectancy significantly mediated the influences of two different hypotheses of two predictors: agreeableness and openness to testing CI. A significant moderating impact of conscientiousness was found on the relationship between performance expectancy and CI and the relationship between social influence and CI. The findings of this study indicated that rigorous attention to the personality of individual nurses and substantial TMS could improve nurses’ CI to use EHRs. A literature gap was filled concerning the mediating effects of performance expectancy on the FFM-CI relationship, and the moderation effects of Conscientiousness on UTAUT constructs and CI are another addition to the literature. The results are expected to assist government agencies, health policymakers, and health institutions all over the globe in their attempts to understand the post-adoption use of EHRs.

## 1. Introduction

There has been tremendous growth in the number of digital health tools, which have the potential to significantly improve the quality and efficiency of healthcare delivery in the last few years. Despite this, the use of these tools in large, complex health systems is still relatively limited. To make digital health tools more accessible to people throughout an enterprise, it is imperative to introduce strategies to address the challenge of digital health tool adoption and implementation; and to help tools move from being validated to being incorporated into workflows for a large health system [1,2].

Despite the increasing interest of large healthcare systems in digital health tools, it is a complex area to grasp [3]. Digital health tools are primarily developed for use within health systems, including many players, already established and entrenched suppliers, and large electronic health record (EHR) systems providers. Numerous regulatory compliance issues and specific barriers exist, such as, financial support, interoperability, and training of technical support staff to successfully adopt new digital health tools within these large complex organizations [1,4].

Due to patient needs and the attendant overhead expenses globally, healthcare systems have experienced increased strain on their physical and monetary resources. Consequently, investments in Information Systems (ISs) to raise the efficiency of medical systems and lower the costs of running healthcare facilities have become a significant area of focus for governments and hospitals. These ISs are used to gather patient information from diverse sources into a single digital archive [5,6,7].

Electronic health records (EHRs) have become the most used means of documenting patient data, including medical appointments, and demand for the software is growing [5,8]. Electronic health records have been acclaimed as a technological breakthrough capable of significantly altering the future of the healthcare industry in terms of improved service delivery and systems quality [9,10,11,12]. Today, Information Technology (IT) is used ubiquitously [13,14,15] and has become paramount for economic development [16]. Countries that cannot afford, access, or mobilize the most advanced technologies cannot compete in an increasingly digitized age.

Nonetheless, the number of benefits that healthcare institutions derive varies highly, mainly due to disparities in EHRs acceptance among hospital staff based on the level of adoption (fully or partially implemented). Ineffective EHRs, or partially implemented ones, will affect clinical performance, users may exhibit negative reactions, the workflow may be disrupted, the further implementation may become neglected, and the initiative may ultimately fail [17]. Hospitals must learn how the system works to achieve greater efficacy, efficiency, effectiveness, and accuracy in their operations. Consequently, it is imperative to know how the EHR works and its characteristics, like its ability to simplify administrative tasks [18]. Therefore, the attitudes and influencing factors of health professionals toward usage of EHRs must be understood to overcome current obstacles to adoption of EHRs and maximize their benefits.

Researchers have used various models and theories in economics and social science to examine the acceptance of information technology (IT) in the healthcare sector. They can be grouped according to the technology deployment types, such as pre- and post-adoption of technologies [19,20], and the healthcare professionals they target like physicians [21], nurses [22], secretaries [23], and the different evaluation dimensions and attributes they are built on.

Over the last 10 years, the concept of the continuance intention to use IT has become a key area of IS research [15,19,24,25]. An IT system cannot be considered successful if users do not sustain its usage [19,26] or the system experiences underutilization and/or abandonment [24,27,28,29]. In this context, past studies have examined the usability, adoption, and usage of EHRs (acceptance studies), along with the intention to continue to use or increase their usage habits (continuance intention) [30,31,32]. Nevertheless, most acceptance and continuance intention studies have focused on pre-or early utilization phases in targeted groups of users such as physicians or single model-based evaluation [24,30,31,32]. The current work context is nurses engaged in the meaningful usage phase of fully integrated EHRs in hospitals.

## 2. Literature Review

The main dependent dimension in the acceptance model is use or usage behavior. In this regard, Davis’s (1985) Technology Acceptance Model (TAM) was developed based on the Theory of Reasoned Action [33] and has been the most extensively utilized model to examine the behavior intention of users [34,35]. The model posits that end-user attitudes function as a determinant of their behavioral intention to use and final actual use, depending on the perceived utility (PU) and perceived ease of use (PEOU) of the IT system. The initial TAM model has been extended many times (e.g., [18,20,36,37,38,39,40]). For instance, TAM2, an extended TAM model, was tested in a longitudinal study testing four systems and examining two of each voluntary use and mandatory use. The findings showed that TAM2 explained 40–60% of the usefulness perceptions variance and 34–52% of the usage intentions variations. In addition, social influence and cognitive factors significantly affected user acceptance.

Moreover, the Unified Theory of Acceptance and Use of Technology (UTAUT) and its different variants that [11,36,38] proposed showed that four main factors influenced technology acceptance: usefulness, social norm, ease of use and facilitating conditions. In a related study, [18] examined the use of e-patient records in acute care settings among nurses and found UTAUT explained 33.6% of use variance and 54.9% of satisfaction among users. Furthermore, in the context of acceptance of mobile Internet (m-Internet), [36] found that UTAUT successfully explained 65% of the behavioral intention variance. Notably, acceptance models can be used in pre- and post-adoption situations (use intention in the former and use behavior and current use in the latter).

In post-adoption contexts, continuance intention models have become popular, with the intention towards continuous use examined on the total or partial deployment of the IT system. The studies [41,42] examined satisfaction (SAT) and perceived usefulness (PU) as intermediary dimensions, while confirmation of expectations (CE) was examined as the independent dimension. Study [41] proposed a direct CE-PU relationship, and in the case of online banking, the model managed to explain 20% of PU variance, 33% of SAT variance, and 41% of CI variance. Other studies (e.g., [43,44]) also supported the explanatory power of the ECT model in various IS fields.

### 2.1. Theoretical Foundation

A few decades ago, scholars proposed a myriad of models and theories to distinguish which factors bore weight in accepting and using IT, especially from the end user’s viewpoint [45,46,47]. The UTAUT model [37], which synthesizes 14 constructs extracted from eight established models and theories on IT acceptance, offers valuable insights [48,49,50,51].

The UTAUT model has proposed constructs that influence the intention to use and the actual use of IT [52]. The four constructs identified are (1) effort expectancy, (2) performance expectancy, (3) facilitating conditions, and (4) social influence. These constructs concentrate on user perceptions of how effectively a system boosts productivity [53]. The same four constructs serve equally well to help define how easily any given person can learn to utilize a new system. Furthermore, the conduct of significant others surrounding them in the workplace can affect the use of IT by individual workers. Similarly, workers’ sense of the resources and assistance they can access in utilizing a new system will influence if and how they use it [37,54].

While the UTAUT can be used to predict a one-time action—that of accepting and using an IT system for the first time, for example, by any given individual—the theory does not account for what happens post-adoption [18,55,56]. Yet, in a behavior determined by the UTAUT process, attitude is examined before behavioral intention and system utilization [57,58]. Measuring a user’s CI to apply the EHR system following their initial utilization would be appropriate for modifying the concept of intention utilized in UTAUT.

The present study builds on this stream of research [18,28,59,60] by examining how the use of IT systems, specifically EHRs, can support the professional activities of users in terms of interpreting the data collected in digitalized records. Accordingly, this study applied the UTAUT model but replaced the dependent variable of intention with CI. This need to do so has been recognized; [55] acknowledged the dearth of understanding and empirical evidence regarding how the UTAUT model can apply to the healthcare sector, highlighting this gap, especially regarding the crucial factors for practitioners to contemplate before accepting new technology. Hopefully, by addressing this gap, the understanding of CI within the UTAUT model structure will be extended.

As [61] noted, not many studies have examined the role of personality in explaining how and why people use technology, linking these with UTAUT to clarify intention [62,63,64]. However, the IT and IS literature [65,66,67,68,69,70,71] noted that “although mounting evidence suggests individual differences influence IT use, more integrative research is needed to better understand the nomological net among individual differences that relate to IT acceptance and use” (p. 382). Consequently, recognizing how and why users accept and use information systems technology is demanded. Against this background of the need to know, theories of UTAUT have proliferated [37,72,73].

Furthermore, employing wrong managerial tactics to facilitate adoption behavior through various stages may have serious consequences, leading to an entire reduction in IS effectiveness [74] or discontinuing system usage. So, Top Management Support (TMS) has an overarching role. Several studies have investigated TMS within the context of information technology use [75,76,77]. The current study incorporated TMS due to its significant role in inspiring the adoption of innovations within healthcare organizations [78]. Top management’s information and knowledge about EHRs diminishes doubts about these innovations [79] and thus encourages healthcare providers to continue using EHRs. According to the best available knowledge, few studies have investigated the effects of TMS on CI in health information technology use.

Previous research has discussed the factors affecting the success of both IS innovations and healthcare IS innovations [80]. The UTAUT and the expectation-confirmation theory were the main theories extensively applied to understand the continuance usage of technological innovations. The five-factor model (FFM) was the predominant theory used to investigate the impact of user personality on IS usage [61]. However, a literature review of this topic reveals three important gaps. The first and the second concern the application of the FFM to examine how user personality affects health information innovations, while the third gap relates to the application of top management support (TMS) as an external variable [24].

The present study responds to the first and second gaps mentioned above by introducing the FFM model to complement the UTAUT model theoretically. In the fields of management and psychology, research has applied the FFM extensively for empirical purposes to anticipate attitudes, behaviors, and perceptions; however, in the field of IS, the model remains under-researched and underused [81,82,83]. Specifically looking at research on health information technology, the personality factor has been largely overlooked [84]. This current study investigates how conscientiousness functions as a moderator variable on the respective relationships of effort expectancy, performance expectancy, facilitating conditions, and social influence with CI to use EHRs. It also examines performance expectancy as a mediator variable on the respective relationships of agreeableness, conscientiousness, extraversion, neuroticism, and openness to experience with CI to use EHRs.

There is limited literature on innovation usage that discusses the role of TMS in the health context. Management’s role in motivating and convincing the healthcare staff about the potential benefits of healthcare information systems (HISs) is critical; the failure of management support in this regard may lead to challenges for adoption and usage [85,86]. Conclusions from related empirical studies also confirm that if management is disoriented and fails to implement a complete strategic framework for HIS implementation, then the realistic goals and objectives set for employees may not be fulfilled [87,88]. Notably, previous studies have affirmed that knowing top management have accurate and substantial information and knowledge of EHRs would diminish employees’ doubts about these innovations [79] and thus encourage employees, specifically healthcare providers, to continue using EHRs.

However, the healthcare system is already complex, and introducing a new system requires that individual roles be changed, that interrelation between and among personnel using the systems be friendly, and that potential users be sufficiently willing to take on a new system. Greenhalgh et al. [89] also highlighted the importance of considering the “socio-technical” aspects of change, ensuring the synchronization of the interrelated technical and social systems. For instance, complexity might discourage workers in the healthcare industry from using new technology because they would be required to attend training programs and spend extra time learning how to use it, so adding further time constraints to their loaded schedules [90,91].

### 2.2. Proposed Model and Hypotheses Formulation

#### 2.2.1. The Relationship between Effort Expectancy and Continuance Intention

Effort expectancy refers to how easy it is for a given IS to be used [37]. The more difficult an innovation is to use, the less likely consumers will adopt it [54,92]. Effort expectancy thus creates a barrier that hinders the utilization of technological innovations. How effort expectancy is perceived depends on an individual’s on-the-job experience [37]. If a person still has no on-the-job experience, then his/her impression of how easy an innovation is to use will be based on his/her opinions about using computers in general. First-hand experience in using new technology can create new perceptions. For instance, a user may expect a particular website to be user-friendly, but actual use confirms or contradicts this expectation. Effort expectancy has been shown to influence intention to use, as well as its indirect effects through attitude [37]. In continuance contexts, this is likely to be true, because human tendencies to subconsciously pursue instrumental behaviors are independent of their timing or stage of such behaviors [41]. Based on the above, the following hypothesis is posited:

**Hypothesis** **1.** *Effort expectancy will positively influence the continuance intention of Jordanian nurses to use electronic health records.*

#### 2.2.2. The Relationship between Facilitating Conditions and Continuance Intention

Facilitating Conditions describes the extent to which an individual believes that an IS can be used as a result of the existence of organizational and technical infrastructure [37]. Such a perception of a potential user is associated with that individual’s ability to control the use of the IS in question. This perception is generally considered to be able to directly affect the intention to use and the usage of IS [37]. People may develop a positive attitude towards a new IS if they have sufficient access to resources that enable and facilitate usage. User perceptions of the required information and support to use an IS may differ before and during usage. If when employees use an IS, the resources at their disposal, like access to equipment or online assistance, are greater than they had expected before using it, a positive sense of facilitating conditions will develop. This positive confirmation induces a greater level of satisfaction and improves facilitating conditions post usage. These engender more positive attitudes to usage and greater CI to use [60].

Regarding the expectation-confirmation theory’s logic [38], positive confirmation of facilitating conditions positively affects post-usage facilitating conditions. Judging by existing research, post-usage facilitating conditions will positively influence CI, indirectly impacting CI through post-usage attitudes [37]. Based on a literature review, Zhou Ref. [59] explicitly acknowledged how facilitating conditions positively affected CI. Thus, the following hypothesis is posited:

**Hypothesis** **2.** *Facilitating conditions will positively influence the continuance intention of Jordanian nurses to use electronic health records.*

#### 2.2.3. The Relationship between Performance Expectancy and Continuance Intention

One definition of performance expectancy is A person’s belief that using the system will assist him or her in improving his or her performance at work [37]. Thus, if nurses foster positive expectations regarding the use of EHRs, it is likely that they will persist in using them. Prior research has demonstrated how perceived usefulness positively impacts continued utilization [93]. Bhattacherjee [41] in his study underlined that perceived usefulness or the expectation that usage will improve performance positively affects the intention to continue using, just as it affects the initial acceptance to use. So, the following hypothesis is posited:

**Hypothesis** **3.** *Performance expectancy will positively affect the continuance intention of Jordanian nurses to use electronic health records.*

#### 2.2.4. The Relationship between Social Influence and Continuance Intention

Research on IS usage acknowledges that social influence is the level of approval a potential user discerns among significant others regarding his/her usage of a new IS [26,94]. This factor is recognized as having a considerable impact on determining intention across many empirical endeavors [95,96]. It has been posited that social influence can directly affect intention. The behavior and opinions generally inform these perceptions of approval before usage and any changes observed in significant others such as friends and peers. Relevant studies have also suggested that this factor will positively influence CI [59,60]. Therefore, the following hypothesis is posited:

**Hypothesis** **4.** *Social influence will positively affect the continuance intention of Jordanian nurses to use electronic health records.*

#### 2.2.5. The Relationship between Top Management Support and Continuance Intention

Top management support (TMS) relates to the recognition by those in the top management of how important an IS can be and their involvement in its application [97]. The study [98] said TMS means the “explicit and active support of the top management towards the introduction and development of new information technology” (p. 138). Multiple studies have testified to the crucial role that TMS plays in increasing an organization’s adoption of technological innovations [99,100,101,102,103,104,105,106].

Looking specifically at the case of EHR systems, the most challenging factors in their implementation are related to the numerous complexities of HISs, including distinguishing and advanced technical characteristics, the complex performance of an administrative role, and multiple issues concerning the safety and security of patients and their data. Introducing a new technological tool to manage a multifaceted system that is by nature intricate and complex, is not a simple task. Added to the mix, is the need to ensure that the change is sustained and that it carries with it numerous different people, including their roles and relationships with each other and an organization’s socio-technical dynamics or business processes [89,107,108]; TMS can make a radical difference in engaging all these elements in such a change. Implementing IS innovations requires considerable material and managerial resources to establish the necessary infrastructure and adequate support mechanisms that users need [84,85,86,87,99,109]. Without TMS, mobilizing these resources is a struggle. Top management support legitimizes the introduction of a new IS, indicates that management is committed to applying it effectively, and helps to convince and motivate staff to adopt it despite any initial challenges in learning how to use it [84,86,110].

As [41] emphasized, the successful establishment of an IS innovation also depends on how well users are supervised and assisted throughout the process. It is the role of management to make the utilization of an IS innovation obligatory and motivate, encourage, and help their employees use it. As [101] noted, managerial support is essential in transforming work-related systems and processes and how they are conceptualized; this is vital for the success of applying a new IS.

To the best knowledge, little research has examined how TMS relates to CI, despite numerous studies indicating that TMS positively affects intention to use [29,75]. Hence, the following hypothesis is posited:

**Hypothesis** **5.** *Top management support will impact the continuance intention of Jordanian nurses to use electronic health records.*

#### 2.2.6. The Relationship between Agreeableness and Continuance Intention Using Performance Expectancy as a Mediator

Agreeableness is an adjective commonly applied to people who demonstrate the ability to understand others. It also implies altruism, compliance, modesty, trustworthiness, a straightforward demeanor, and tender-minded character [111]. Other traits include being considerate, cooperative, kind, likable, and willing to help [112].

Prior research has indicated that agreeableness can be a valid predictor for taking on jobs requiring interpersonal interaction and teamwork, particularly in cases where this interaction includes assisting and/or working closely with others [113]. Thus, when a technological innovation encourages working together with other people to effectively achieve job tasks, agreeable employees will likely be interested and motivated to use it. Compared to other personality types, agreeable individuals are usually more willing to try and more likely to comply with a request to utilize new technology. Agreeable types are more likely to have a greater interest in the cooperative elements of innovation than the promise to facilitate performance [83]. Therefore, the following hypothesis is posited:

**Hypothesis** **6.** *The relationship between agreeableness and the continuance intention of Jordanian nurses to use electronic health records will be mediated by performance expectancy.*

#### 2.2.7. The Relationship between Openness to Experience and Continuance Intention Using Performance Expectancy as a Mediator

Openness to experience is defined by how ready and willing an individual is to learn something new or engage with new ideas. Individuals with a high level of openness to experience tend to seek out the unfamiliar and relish change [83]. Such people will typically respond positively to and engage easily with technological advances newly introduced into the workplace; they will be unlikely to feel the apprehension that other personality types might experience in the face of new technology.

Openness to experience is born of the desire to do something different, and an individual’s approaches to and use of new technology will reflect this attitude. As per behavioral decision theory, people are usually prone to seek and highly value information that aligns with their ideas and aspirations while disregarding information that does not [114]. This process is termed confirmatory bias [115]. Accordingly, people who are more open to experience tend to give greater weight to reasoning that aligns with their keenness to try out the unfamiliar and would likely be more easily convinced by arguments that support the long-term usage of advanced technological innovation. Thus, the following hypothesis is posited:

**Hypothesis** **7.** *The relationship between openness to testing and Jordanian nurses’ continuance intention to use electronic health records will be mediated by performance expectancy.*

#### 2.2.8. The Relationship between Neuroticism and Continuance Intention Using Performance Expectancy as a Mediator

Neuroticism is defined as an individual’s propensity to suffer psychological distress [116]. It typically encompasses feelings of timidity, low confidence, guilt, and stress. Neuroticism often hosts anxiety, depression, aggression, impulsiveness, self-consciousness, and susceptibility to stress [111].

Neuroticism typically engenders a negative perspective on all aspects of life. Highly neurotic individuals tend to categorize any given stimulus negatively and respond accordingly, while less neurotic individuals might identify that same stimulus as a positive [83]. Therefore, the assumption can be made that such an attitude would likely manifest in a neurotic individual’s performance expectancy of a given technology. Indeed, it is probable that technological innovations in the workplace elicit negative feelings of intimidation and apprehension among those with neurotic personalities. Notably, technology at work often enables monitoring each person’s work, which can cause additional worry to neurotics, further aggravating their negative attitude toward introducing and using that technology. Thus, the following hypothesis is posited:

**Hypothesis** **8.** *The relationship between people who own a low level of neuroticism and the continuance intention of Jordanian nurses to use electronic health records will be mediated by performance expectancy.*

#### 2.2.9. The Relationship between Extraversion and Continuance Intention Using Performance Expectancy as a Mediator

Extraverts are always active, warm, frank, bold, excited, and positive [111] and are outgoing, social, and value close interpersonal relations [117]. Research has claimed that extroverted individuals perform especially well in professional positions requiring strong social skills, such as sales and management [118]. Extraverts also tend to be significantly concerned with their social status—how they represent themselves to others and how others perceive them.

In general, a key incentive behind an individual’s adoption of new technology is the hope of improving his/her social status [119]. To create or uphold a positive image in the eyes of others who are important to them, a person will often adopt a behavior they assume those others approve of [96]. The subsequent increase in that person’s power and influence is likely to indirectly enhance his/her job performance as he or she attempts to maintain the image of professional improvement and elevated social status. Given their concern with how others perceive them and their behavior, it is likely that extraverted individuals demonstrate CI to use technological innovations as they attempt to maintain the good opinions of those around them. Thus, the following hypothesis is posited:

**Hypothesis** **9.** *The relationship between extraversion and Jordanian nurses’ continuance intention to use electronic health records will be mediated by performance expectancy.*

#### 2.2.10. The Relationship between Conscientiousness and Continuance Intention Using Performance Expectancy as a Mediator

Conscientiousness combines a person’s keen sense of responsibility, the impetus to succeed, self-discipline, proficiency, and reliability [111]. Conscientious personality types have an innate desire to accomplish and deliver optimal performance; they demonstrate a constant readiness to implement any changes that will improve their performance. Self-discipline is a critical aspect of their character, indicated by their ability to meet their objectives and persistent motivation to aim higher [120,121]. The management literature has recognized the construct of conscientiousness for its vital significance concerning job success [118].

Conscientious individuals tend to carefully evaluate the extent to which technological innovation will offer the chance of ever more professional achievements before accepting to use it or not. Typically, a conscientious person will commit to and persist in acting on his/her intentions. Thus, the following hypothesis is posited:

**Hypothesis** **10** . *The relationship between conscientiousness and Jordanian nurses’ continuance intention to use electronic health records will be moderated by performance expectancy.*

#### 2.2.11. The Relationship between UTAUT Variables and Continuance Intention Using Conscientiousness as a Moderator

Our literature review revealed a lack of research concerning the moderating effect of the five-factor model (FFM) on the CI to use technology and the continuance usage of technology. So far, studies [81,83,122,123,124] have been restricted in investigating the moderating effect of FFM on the relationship among the UTAUT variables like effort expectancy, performance expectancy, facilitating conditions, and social influence and intention to use. No available studies have investigated how the FFM meditates the relationship between UTAUT variables and the CI to use technology. This current study thus builds on previous research to examine conscientiousness (one of the FFM’s five personality domains) as a moderator in the relationship between UTAUT variables and the CI to use technology.

Conscientious individuals distinguish themselves in planning, organizing, and completing their job tasks and are meticulous in their work, punctual, and dependable. Such an individual may demonstrate obsessive behaviors, including being workaholic or compulsively neat. People displaying low conscientiousness tend to maintain lower moral standards and are less vigilant in pursuing their goals [125]. The core trait of a conscientious individual is the self-discipline exhibited in the determination to accomplish, commitment to order, and perseverance [121]. Notably, these latter are essential elements of intrinsic motivation and top performance [126].

The EHRs were introduced to the Jordanian healthcare system based on the government’s belief that EHRs can augment the standard and efficiency of service delivery in the healthcare sector and increase the output and performance of healthcare practitioners. Therefore, the moderating effect of conscientiousness is expected to perform a crucial role in the relationship between UTAUT variables and CI.

#### 2.2.12. The Relationship between Continuance Intention and Effort Expectancy Using Conscientiousness as a Moderator

Extant literature suggests that conscientiousness moderates the relationship between effort expectancy and CI. Individuals with a high level of conscientiousness work hard and can achieve optimal results even if the technology required to accomplish the task is complicated and challenging to use [83]. Therefore, it is likely that how hard or easy a technology is to employ will not affect their usage or performance. However, less conscientious individuals, who do not perform as well consistently, may be concerned about how complex a new technology is and the level of effort needed to utilize it successfully. Until this paper, no research has examined conscientiousness as a moderator of the relationship between effort expectancy and CI. Thus, the following hypothesis is posited:

**Hypothesis** **11.** *The relationship between effort expectancy and the continuance intention to use electronic health records will be moderated by conscientiousness.*

#### 2.2.13. The Relationship between Facilitating Conditions and Continuance Intention Using Conscientiousness as a Moderator

The conscientiousness factor could have influenced how facilitating conditions relate to CI. Individuals defined as conscientious work hard and perform well provided they have the necessary organizational and technical assistance. Thus, highly conscientious employees are likelier to have greater CI to utilize a new technological system than their less conscientious colleagues. Conversely, if an employee believes that his/her company has failed to provide adequate support to enable the use of a new system, a high level of conscientiousness will strengthen their feelings of frustration and/or disappointment and lead to a decline in their CI to use [120]. Our review reveals no research that has explored whether conscientiousness moderates the relationship between facilitating conditions and CI. Hence, the following hypothesis is posited:

**Hypothesis** **12.** *The relationship between facilitating conditions and the continuance intention to use electronic health records will be moderated by conscientiousness.*

#### 2.2.14. The Relationship between Performance Expectancy and Continuance Intention Using Conscientiousness as a Moderator

It has been argued that dispositional tendency significantly impacts how much performance expectancy can elicit the CI to utilize technological innovations. According to [83], highly conscientious individuals tend to be cautious in considering how using technology can enhance their efficiency and performance. If a person perceives that technological innovation can benefit his or her performance, the more conscientious that person is, the more he or she will develop the CI to use an innovation. Conversely, in someone who perceives no use in an innovation, conscientiousness will magnify the impression of its uselessness and thus reduce the individual’s CI to use it [83]. No other study seems to have tested how conscientiousness moderates the relationship between performance expectancy and CI. Thus, the following hypothesis is posited:

**Hypothesis** **13.** *The relationship between performance expectancy and the continuance intention to use electronic health records will be moderated by conscientiousness.*

#### 2.2.15. The Relationship between Social Influence and Continuance Intention Using Conscientiousness as a Moderator

Conscientiousness is assumed to bear weight on the relationship between social influence and CI. The authors [83] claimed that individuals with a high level of conscientiousness are careful in considering their opinions on whether to persist in using technological innovation. Thus, the perception that important individuals around them support the continued use of a given innovation will likely secure strong CI to use it among conscientious people. Furthermore, conscientious individuals assess and consider negative social influence, which is the perception that important others do not support the use of a given innovation. Therefore, negative social influence negatively affects the CI to utilize a new technology among conscientious individuals, whereas it will have less of a bearing on the CI of those with less conscientiousness who take less account of others’ points of view. Our literature review revealed no studies exploring how the personality trait of conscientiousness impacts how performance expectancy relates to CI. Thus, the following hypothesis is posited:

**Hypothesis** **14.** *The relationship between social influence and the continuance intention to use electronic health records will be moderated by conscientiousness.*

**Figure 1:**
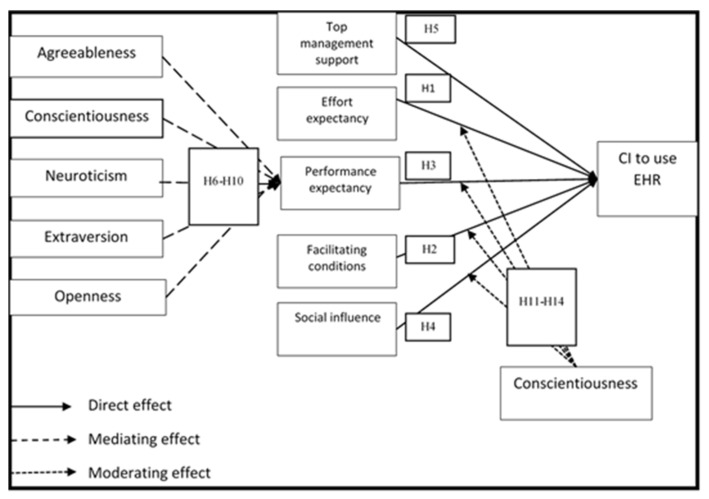
Theoretical Framework of the Relationship between the FFM, UTAUT Variables, Top Management Support, and Continuous Intention (CI) to Use Electronic Health Records (EHRs).

An overview of the theoretical framework for the relationship between the FFM, UTAUT Variables, Top Management Support, and Continuous Intention to Use Electronic Health Records (EHRs) is presented in Figure 1.

## 3. Methodology

### 3.1. Research Context

Across all sectors, the use of IT is transforming operations. In more economically developed countries, IT has been integrated extensively into all areas of the economy and society, the healthcare sector included. Less economically developed countries lag in its rollout and usage [127,128]. Arab nations are among those that generally lag in the effective use and application of IS technology in healthcare facilities; notably, state hospitals use IT to a very limited extent [24,129,130].

Jordan’s healthcare sector is no exception. Besides the inadequacy of its ISs, Jordanian clinics and hospitals also fail to provide high quality service due to inaccessibility, poor resource use, and lack of effective management [22,131,132,133]. Even when an EHR system is put in place, it is often ineffective because EHR systems are often challenging to navigate. As a result, the positive impact that EHR systems are supposed to produce, including lightening the load of work for healthcare providers, never materializes. Indeed, the challenge of learning how to use EHRs and use them well might even diminish practitioners’ performance and productivity overall [134]. Several studies have demonstrated that healthcare professionals consider EHR systems frustrating, laborious, hard to utilize, and potentially inadequate in assuring patients’ privacy [135,136]. Such negative perceptions will likely lessen users’ continuance intention (CI) to employ an EHR system. Ultimately, users could reject using EHRs entirely in favor of traditional paper documentation.

In Jordan, the CI to employ an EHR system among health practitioners, especially nurses, is considered deficient [24,131]. However, definitive information on this issue is missing, a debilitating knowledge gap for the Jordanian government, which thus has no concrete idea about the extent of EHR usage despite the millions spent to facilitate this system in the healthcare industry [24,131]. Hence, investigating the acceptance and use of new technological systems such as the EHR in this specific context is necessary. In doing so, the present study addresses the gap in research on the CI of nurses to use EHR systems in Jordan.

### 3.2. Target Population

The population targeted in the current study consisted of nurses employed in public sector hospitals in Jordan who were authorized to use an EHR system. This population was selected because scant theory-based research had hitherto been conducted on nurses’ acceptance and utilization of technological innovations [137]. Jordan’s Ministry of Health categorizes nurses into three groups: first, registered nurses and midwives; second, practical nurses; and third, portal nurses, and authorization to operate EHRs is restricted to registered and practical nurses only. Exclusively, nurses from categories one and two were thus involved in this study. For the first stage, the sampling frame was derived from a list of emails compiled by Electronic Health Solutions Company in Jordan that represented public hospitals with a fully operational EHR, and for the second stage, a list of nurses who work in those hospitals.

### 3.3. Sampling Procedure

The primary sampling unit of this research was Jordan’s 10 public hospitals that comprehensively operate an EHR system, and the secondary unit was individual nurses authorized to utilize that system. Jordan’s 11 public hospitals with a fully operational EHR were identified, but the King Hussein Cancer Center was unable to participate, leaving 10 participating institutions. Given that this figure was so small, the study used all the remaining 10 hospitals. Department heads from each hospital provided lists of the nurses working in their hospitals, and the sample population comprised 2194 nurses spread over the 10 hospitals. According to [138], the sample size must fall between 322 and 327 for a study whose population is of this size. The following formula was used to calculate the number of respondents needed from each hospital: (number of nurses in hospital *x*/total number of nurses in all hospitals) × sample size (refer to Table 1). To guarantee a minimal sample response of 327, the researchers distributed 510 questionnaires. These questionnaires were distributed in person by applying systematic random sampling, a probability sampling procedure, nurses employed in these 10 hospitals were selected: heads of departments provided lists of the nurses working in their respective hospitals, and following every fifth name, one name was selected at random. Of the 510 questionnaires distributed, 490 were returned; 18 were excluded because 50% of the information required was lacking [139]. Thus, the final number of questionnaires used in the study was 473, reflecting an effective response rate of 92%. See Table 2.

### 3.4. Constructs and Their Measurements

This study employed constructs and the associated items previously tested in other studies to ensure discriminant and convergent validity. These included six items adapted from [141] to represent CI; five items adapted from [137] to represent performance expectancy; five items adapted from [137] to represent effort expectancy; eight items adapted from [137] to represent social influence; six items from [137] represent to facilitating conditions; and eight items adapted from [142] to represent TMS. Finally, the NEO-FFI measurement [125] was utilized concerning personality. This comprised 60 items, 12 items for each dimension (neuroticism, extraversion, openness, agreeableness, conscientiousness), which are well-validated measures of the big five personality factors that have been utilized widely in previous research [83,143,144]. Several items were altered to ensure their appropriateness in the specific cultural context.

All variables employed were clearly defined and described in the questionnaire to guarantee that the questionnaire items were unambiguous and that the responses were accurate. The definition used for CI was based on [41], which was the user’s intention to progress using an IS post-adoption. To be precise, CI represented the intention of nurses in Jordanian public hospitals to progress to use EHRs post-adoption. The questionnaire was translated from English into Arabic according to [145]’s guidance. Before the study, four academics with a high level of IT knowledge evaluated the questionnaire. The questionnaire was subject to a few minimal modifications following this pre-test to enhance the clarity of the questions. All questionnaire items used a 5-point Likert-type scale (i.e., 1 = strongly disagree, 5 = strongly agree).

## 4. Results

The model proposed was tested using the partial least squares (PLS) regression analysis. The PLS analysis is a structural equation modeling technique that evaluates the measurement and structural model [146,147]. The initial stage in the PLS process involves testing the measurement model regarding reliability and validity [148]. Reflective measurement models are generally assessed in terms of four factors: indicator reliability, internal consistency (composite reliability), convergent validity (average variance extracted [AVE]), and discriminant validity. The composite reliability coefficients of the study’s constructs ranged from 0.792 to 0.951. Thus, the measures employed appear to have sufficient internal consistency reliability [149,150,151]. The AVE of all constructs scored above the conventional value of 0.5 (Table 3).

Discriminant validity was evaluated using the AVE square root calculated for every construct; all square roots were greater than the correlations among the constructs, proving discriminant validity (Table 4).

The main effect model and the mediating effect where the moderator was excluded were measured to assess the structure model and test the proposed hypotheses. The interaction model was subsequently used to check the moderation effect [150,152]. The PLS path algorithm used to generate the path coefficient was applied to evaluate the significance of the different effects of the study model. The structural model was then applied through the bootstrap procedure that generated 500 resamples [150,153,154]. Finally, the significance of the path coefficient was evaluated in three segments: direct effect (Table 5), mediating effect (Table 6), and moderating effect (Table 7).

Table 5 shows that, in line with the study hypotheses, a significant and positive influence (β = 0.314, *t* = 5.284, *p* < 0.01) on the CI of Jordanian nurses to use EHRs was found by effort expectancy. The H1 was therefore upheld. The impact of facilitating conditions was also positive and significant (β = 0.091, *t* = 2.246, *p* < 0.02) on the CI of Jordanian nurses to use EHRs, thereby supporting H2. Performance expectancy significantly and positively (β = 0.491, *t* = 10.425, *p* < 0.01) affected the CI of Jordanian nurses to use EHRs. Hence, H3 was confirmed. Results for social influence were positive and significant (β = 0.075, *t* = 1.756, *p* < 0.05) in terms of its relationship with the CI of Jordanian nurses to use EHRs, upholding H4. In contrast, a negative and insignificant correlation (β = −0.067, *t* = 1.515TMS) was found with TMS and showed no influence on the CI of Jordanian nurses to use EHRs. H5 was therefore rejected.

To test hypotheses H6 to H10, the mediator analysis procedure of [155] was used to assess the mediating influence of performance expectancy on how personality dimensions relate to CI (see Table 6). As [155] suggested, if the indirect effect is significant and the bootstrapped confidence interval (Boot CI) did not straddle a 0 in-between, this indicates mediation. The bootstrapping analysis for agreeableness demonstrated a significant indirect effect (β = 0.104) with a *t* value of 2.958. The indirect effect 95% boot CI: (LL = 0.034, UL = 0.171) did not straddle a 0 in-between, suggesting mediation [155]. For openness to experience, the bootstrapping analysis demonstrated that the indirect effect (β = 0.092) was significant with a *t* value of 2.575. Again, mediation was implied in that the indirect effect 95% boot CI: (LL = 0.020, UL = 0.161) did not straddle a 0 in-between. These results thus offer evidence that performance expectancy plays a mediator role in the relationship between agreeableness and CI and between openness to experience and CI, thereby upholding hypotheses 6 and 7. However, performance expectancy did not significantly mediate the relationship between neuroticism and the CI to use, extraversion and the CI to use, and conscientiousness and the CI to use. Hence, hypotheses 8, 9, and 10 were all unsupported.

**Figure 2:**
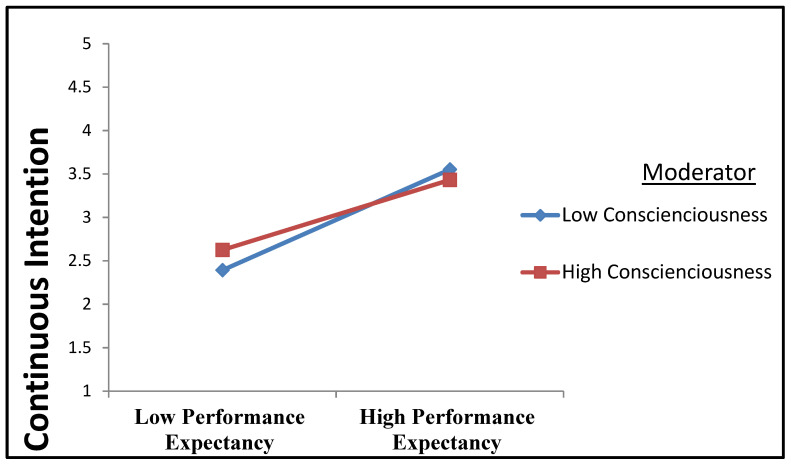
Conscientiousness Dampens the Positive Relationship between Performance Expectancy and Continuous Intention.

An interaction model was applied to examine hypotheses H11 to H14 regarding conscientiousness as a moderator. Four latent interaction constructs were created to depict the interaction between predictors (UTAUT-related factors) and conscientiousness on the criterion variable (CI to use EHRs). The model was subsequently tested using a bootstrapping procedure with 500 resampling. Table 7 details the results. Apart from the interaction terms between conscientiousness and performance expectancy and conscientiousness and social influence, all interaction terms’ path coefficients were relatively weak (less than 0.1) and insignificant (*p* > 0.05). The negative moderation path coefficient of the interaction term between conscientiousness and performance expectancy (β = −0.097, *t* = 2.021, *p* < 0.05) indicates that conscientiousness had a negative moderating effect on the relationship between performance expectancy and the CI to use EHRs. The H13 was thus confirmed. Data from the path coefficients highlighted the influence of conscientiousness as a moderator on the relationship between performance expectancy and the CI to use as per the guidelines set down [156,157]. Figure 2 illustrates how conscientiousness works as a moderator to manage the relationship between performance expectancy and the CI to use the EHRs in that conscientiousness diminishes the positive relationship between performance expectancy and CI.

**Figure 3:**
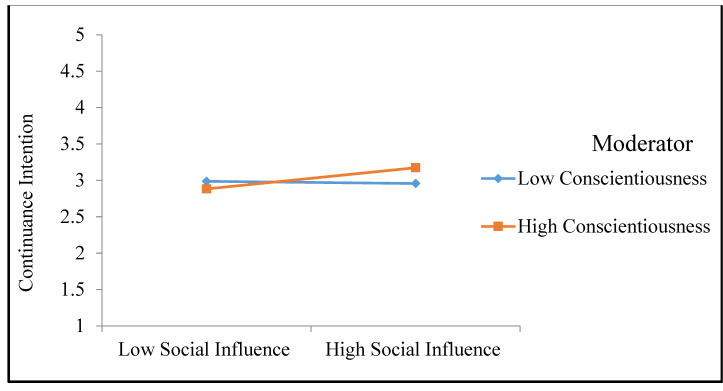
Conscientiousness Strengthens the Positive Relationship between Social Influence and Intention.

Furthermore, the positive moderation path coefficient of the interaction term between conscientiousness and social influence (β = 0.095, *t* = 2.133, *p* < 0.05) offers evidence that conscientiousness positively moderates the relationship between social influence and the CI to use EHRs. The H14 is hence upheld. Figure 3 illustrates this result, portraying how conscientiousness acts as a moderator in the relationship between social influence and the CI to use, in that it reinforces the positive relationship between social influence and CI. In addition, the findings demonstrate that when the interaction terms are included, the model productivity is improved. Here, the R-square increased from 0.696 to 0.700.

As a final consideration in evaluating the PLS structure model, the last criterion is its coefficient of determination (R^2^). Accordingly, researchers such as [153] describe the general rule of thumb regarding (R^2^) as values of 0.67, 0.33, and 0.19 are considered substantial, moderate, and weak, respectively. In this study, researchers found that the integration and extension of the UTAUT, expectation-confirmation theory and FFM models results in a successful model for predicting nurses’ Continuance Intention to use electronic health records. With the addition of variables to the integrated model, this model has high predictive power. Researchers found that the study’s model could explain 0.70 of the variance of nurses’ Continuance Intention to use EHRs and 0.19 of the variance of nurses’ performance expectancy.

## 5. Discussion

This research applied a theoretical framework rooted in the UTAUT, expectation-confirmation theory, and FFM to investigate the Continuance Intention to use electronic health records (EHRs) among nurses in Jordanian public hospitals. The study proposed performance expectancy as a mediating variable on the relationships between the different personality dimensions and CI, specifically suggesting conscientiousness as a moderator. Although some covariates emerged as insignificant, the results of this study confirm the predictions of the UTAUT, expectation-confirmation theory, and FFM.

The data analysis revealed that effort expectancy was positively and significantly related to the CI to use EHRs. This finding aligns with the UTAUT, which posits that effort expectancy directly determines technology usage and intention to use [158]. This result can likely be explained by the fact that nurses use most of their working hours to carry out one-on-one patient care activities such as providing treatment, administering drugs, and conducting clinical evaluations. The level of effort required for use will affect nurses’ utilization and intention to use EHRs. Consequently, a user-friendly interface for EHRs is a crucial point on which to focus in developing EHRs.

Furthermore, the findings indicate that facilitating conditions such as the availability and accessibility of the required resources and knowledge positively influence the CI of nurses to use EHRs after the initial utilization in alignment with the UTAUT [60]. In this study, nurses expressed the belief that they had the resources and knowledge necessary to continue using the EHR system in place.

Furthermore, the findings confirm the role of performance expectancy on CI to use technology. Performance expectancy was revealed to be positively and significantly related to CI to use, which once again aligns with the tenets of UTAUT [60] in that higher performance expectancy among nurses elevates their CI to use EHRs. The implication is that nurses are keen to consider how EHRs can facilitate their work and enable them to perform more effectively.

Social influence also significantly and positively affected nurses’ CI to utilize EHRs in Jordanian public hospitals. This result aligns with the predictions of UTAUT but contradicts past research [47], which concluded that social influence does not influence CI. The results indicated that the greater the social influence of other nurses, the stronger a nurse’s intention to continue their usage of the EHR would be. Therefore, the perceptions of significant others regarding the value of the EHR system are vital in determining nurses’ behavior; nurses are concerned about what other nurses think of them. Considering that most nurses in this sample were female, there may well be gender-related issues around the factor of social influence. The study [159] proposed that this impact of social influence was typically strong among women and noted that “women consider inputs from several sources when making technology adoption and usage decisions” (p. 129).

Top Management Support was a key variable in this research. However, the findings imply that TMS did not significantly influence nurses’ CI to use an EHR, contradicting the findings of previous works on TMS and intention to use, which is the nearest concept to CI [77,99,160,161,162,163,164]. This result is because nurses’ TMS and training were deficient before using an EHR. This deficiency was evidenced, first, by the limited duration of the training on how to use the system that nurses received from the EHR provider, Electronic Health Solutions (five days only); second, by not including nurses in decision-making regarding the workflow process as recommended in the establishment of an EHR system. Thus, closing the knowledge gap of nurses with IT and involving them in implementation, training, system design, and IT support processes did not occur. Such involvement can help to stimulate a higher level of interest, engagement in, and commitment to using a new system and indeed in continuing to use it long-term [24]. However, top management in Jordan’s public hospitals failed to offer this involvement, thereby losing a valuable opportunity to generate positive engagement, a sense of ownership, and subsequent motivation among their employee nurses. Nurses generally harbored negative perceptions of TMS. Last, offering incentives and compensation to enhance motivation was absent in the hospitals studied. Hence, the above finding was primarily based on a general lack of TMS within Jordan’s public hospitals, making it impossible for TMS to be a variable capable of impacting EHR usage.

Five hypotheses (H6, H7, H8, H9, H10) were proposed to test the mediator role played by performance expectancy concerning how the five exogenous variables (agreeableness, openness to experience, neuroticism, extraversion, and conscientiousness) related to the one endogenous variable (CI). The findings revealed that both agreeableness and openness to experience were influential in mediating that relationship, confirming H6 and H7. On the other hand, the other three personality domains had no significant impact, thus disconfirming H8, H9, and H10.

Hypothesis 6, which posited that performance expectancy mediates the relationship between agreeableness and the CI to use EHRs among nurses in Jordan’s public health sector, was upheld. Nurses who are agreeable are often accommodating and cooperative in contemplating the utilization of a technological innovation, and they tend to closely adhere to operational and procedural processes [165], including newly introduced systems and mechanisms. Agreeable individuals typically focus on the positive aspects of such innovations instead of any elements that might hinder performance [83] and have high performance expectancy. Among nurses in Jordan’s public sector hospitals, this agreeability ultimately fuels their CI to use EHRs.

Hypothesis 7 proposed that performance expectancy mediates the relationship between openness to experience and CI. The hypothesis was confirmed. Openness to experience was also shown to bear a strong and direct influence on the CI of nurses to use EHRs, a result that echoes previous studies that claimed a significant correlation between openness to experience and intention to use, intention to use is the closest variable to CI [83,166]. The findings demonstrate that nurses who are open to experience actively seek fresh information and ideas with a keen intellectual interest and curiosity. They desire and thus make more of an effort to try out new activities and/or behaviors, such as using an EHR, reflecting research results from various other contexts [167,168,169,170,171].

Hypothesis 8, proposing that performance expectancy plays a role in mediating the neuroticism-CI relationship, was rejected. Individuals defined as neurotic are strongly inclined to feel stressed, nervous, anxious, hopeless, paranoid, and depressed [172], which is likely to translate to a negative perception of and attitude towards the value of technology [83]. However, unlike in other occupations, nurses are typically exposed to numerous health and safety hazards related to the demanding nature of their working environments, the high likelihood of working in unsafe areas, and the high risk of violence [173]. They are frequently required to switch suddenly from a state of boredom to one of maximum attentiveness, having to mobilize in seconds all their energy; they are constantly exposed to people enduring pain and/or distress. Invariably, this places them under considerable emotional, psychological, and physical stress. These working conditions have become a part of nurses’ every day; therefore, nurses, including those who could identify as neurotic, have developed various coping mechanisms to accomplish their duties despite their highly stressful working environment. Thus, neuroticism has minimal impact on a nurse’s professional behavior, and performance expectancy was not able to significantly mediate the relationship between nurses’ CI to use EHRs in Jordan’s public health sector and neuroticism.

Hypothesis 9, which posited that performance expectancy would mediate the relationship between extraversion and CI, was rejected. This finding is consistent with [61], who found that extraversion did not influence the intention to use (intention to use being the closest variable to CI). This current study offers evidence that more extroverted nurses are less likely to welcome the introduction of new technology like EHR, whose usage involves far more individualistic and less communal work than nurses’ traditional tasks. An EHR system demands that more time is spent on documentation and less on patients, consequently leading—in the point of view of nurses defined as extraverted—to decreased performance, lower quality of health care, and less social interaction with other nurses and patients. Thus, extraversion did not significantly influence the CI of nurses to use EHRs, and performance expectancy was demonstrated to have no mediating influence on the relationship between extraversion and CI.

Surprisingly, H10, which posited that performance expectancy has a mediating effect on the relationship between conscientiousness and CI, was rejected. This finding can perhaps be explained as follows. Time is a precious and limited resource for nurses [174]. More time spent on documentation equals less time for patients, resulting in a decline in the quality of care. Presently, nursing schedules are tightly congested: besides large numbers of patients, nurses are tasked with managing the more multifaceted and sophisticated record-keeping demands. The prospect of additional responsibilities in an already frantic day is intimidating. Nurses work under pressure to execute their duties within tight time frames and using technology that is not seen as user-friendly can become time-consuming. In that case, nurses no longer have the time or energy to reflect on their performance or contemplate how to improve it under these circumstances due to the excessive workload and constant stress. To some extent, nurses believe that they can be diverted from their core nursing responsibilities by related administrative duties and often refuse to continue to use a system, not because of the system itself but because they oppose the additional workload. The nurses sampled believed that EHRs inhibited their ability to perform well. Thus, performance expectancy did not play a mediating role in the relationship between conscientiousness and CI.

In addition, another four hypotheses (H11, H12, H13, H14) concerning the moderating influence of conscientiousness on the respective relationships among facilitating conditions, effort expectancy, social influence, and performance expectancy on the one hand and CI on the other were tested. Hypothesis 13 and H14 were confirmed, and H11 and H12 were not.

Hypothesis 11, which posits that conscientiousness bears a moderating influence on the relationship between effort expectancy and the CI to use the EHRs, was not supported. This finding signifies that Jordanian nurses of varying levels of conscientiousness did not experience significantly different impacts on how their effort expectancy related to their CI to use EHRs. A plausible reason for this result is that conscientious individuals tend to be naturally poised to achieve anything they set out to do. Their performance levels are usually high despite the level of effort they expect to have to put in. In addition, they take actions to improve their job performance, for instance, learning how and committing to use EHRs. Hence, in deciding how to respond to the introduction of an EHR system, conscientious nurses were more inclined to thoroughly evaluate the likelihood that EHRs would enable and facilitate greater effectiveness in their work than to focus on determining how easy the system is to use. These nurses gave priority to improving their job performance. Thus, conscientiousness was found not to play any moderating role in the relationship between effort expectancy and the CI to use EHRs.

Hypothesis 12, which posited that conscientiousness would moderate how facilitating conditions relate to the CI to use EHRs, was not supported. One explanation is that conscientious people tend to function at a high level and do whatever is required to better their performance, for instance, using EHRs. In this study, conscientious nurses perceived that their hospitals did not provide sufficient resources and information to use the EHR system effectively and thus enhance their performances. The training provided was poor, and the hospitals did not have enough devices or a great enough variety of devices for everyone to access the EHRs, and the EHR system required large amounts of time to complete all the documentation. Thus, conscientious nurses were dissatisfied and frustrated about the resources and knowledge provided, which did not permit them to perform at a high level. Hence, the relationship between the facilitating conditions and their CI to use the EHR system was comparable to that of their less conscientious colleagues, who were discouraged from using the EHRs given the lack of facilitating conditions.

Hypothesis 13, which posited that conscientiousness would moderate the relationship between performance expectancy and the CI to use EHRs, was supported. Conscientiousness was predicted to play an amplifying role in performance expectancy regarding the latter’s impact on the CI to use EHRs. The results showed that the moderating effect of conscientiousness negatively influenced the relationship between performance expectancy and the CI to use EHRs. This could be because conscientious nurses believe that the application of the EHR system has not helped them improve their job performance. Nurses are typically short of time to complete their daily tasks, and they believe that the use of clinical technology would take too much time, and the lack of a sufficient number of devices providing access to EHRs would prevent them from performing at a higher degree and would not enhance their job performance. Thus, conscientiousness negatively influenced the relationship between performance expectancy and the CI to use EHRs.

Hypothesis 14, which posited that conscientiousness would have a moderating effect on the relationship between social influence and the CI to use EHRs, was supported. Conscientiousness combines with social influence in determining an individual’s level of the CI to use EHRs. Conscientious nurses carefully considered the perspectives of their colleagues as they weighed up whether or not to use EHRs. They reasoned that most of their fellow healthcare workers believed EHRs should be employed, thus developing a stronger CI to use the EHRs. Table 8 summarize the study results.

## 6. Conclusions

Providing high quality and safe care is challenging for health care leaders, decision-makers, and providers. The electronic health records can have an impressive impact on improving healthcare organizations’ efficiency and effectiveness. To achieve this, EHR systems must be used correctly; proper use can minimize patient waiting times, monitor drug side effects, and even decrease mortality rates. In addition, diagnostic and medical errors are reduced, resources are utilized optimally, data quality is improved, and electronic data exchange is more efficient. Overall, this improves user satisfaction and supports healthcare workers in making appropriate medical decisions.

This study highlighted the importance of human personality and TMS in explaining the UTAUT model further. The results revealed a significant positive relationship between effort expectancy, performance expectancy, facilitating conditions, and social influence with the Continuance Intention to use EHRs. However, the relationship between top management support and the CI to use EHRs does not appear to be significant. Additionally, the study revealed that performance expectancy significantly affects two separate hypotheses: agreeableness and openness to experience on the CI to use EHRs. In addition, the study indicated that conscientiousness moderates the relationship between performance expectancy and the CI to use EHRs and the relationship between social influence and the CI to use EHRs.

Moreover, the study emphasized the importance of providing substantial and good-quality training and technical support besides ensuring that the IS itself meets the requirements of the work, notably in operating on a long-term basis to ensure that patients’ records can be kept throughout the course of care. If they thoroughly understood and indeed believed in the practical benefits of EHR systems, nurses would be far more likely to continue to use these systems. Thus, management and nursing supervisors must provide a system that works and works well while ensuring nurses have the knowledge and technical support needed to use it well. Thus, Nurses must possess the necessary attitude and skills to confidently act to bring about necessary changes within their practice environment

### 6.1. Theoretical Implications

This study was conducted to develop a more profound knowledge of how personality, top management support, and nurses’ perceptions affect the post-adoption of EHR systems. It has highlighted the importance of human personality and TMS in explaining the UTAUT model further and extends knowledge on how human personality factors link with and intercede between variables in the UTAUT model. This study also fills the literature gap regarding mediating effects of performance expectancy in the relationship between FFM and CI using UTAUT. Also, it fills the literature gap regarding moderating effects of conscientiousness in the relationship between EE, PE, SI, FC, and CI.

### 6.2. Practical Implications

The findings of this study offer several key practical implications for those responsible for controlling the healthcare sector, healthcare institution managers, government agencies, HIS consultants or vendors, and hospitals. The outcomes are relevant to introducing technology into the healthcare industry at the individual and organizational levels, a process that has the potential to engender significant advancements in the quality of healthcare systems.

In particular, without top management encouraging and assisting employees to use a HIS on a continual basis, the adoption and subsequent usage of that HIS may not transpire. If employee needs for support in using the system to complete or complement the execution of their work are left untended, usage of that system will likely be discontinued. The importance of managerial attitude to EHR implantation and associated behavior towards staff is a significant challenge for managers responsible for enforcing IS innovations in designing a well-organized strategy. Managers must consider the basic tenet that actions speak louder than words: how top managers behave, particularly in terms of their interactions with staff, is directly related to the effectiveness of EHR adoption. The actual transformation of employee behavior and vision sharing among the team depends on managers’ practical, active, relational behavior. So that staff integrates management’s strategic vision with their individual performance goals, top managers should constantly demonstrate support and readiness to assist, allowing employees to share their observations and experiences and actively respond to any issues or queries. Support from top managers is routinely recognized as critical for reconceptualizing work processes and transforming current processes; it is, therefore, a fundamental factor in accomplishing nurses’ sustained use of EHRs.

Another practical implication of this research is the advantage of understanding the kind of individuals who are likely to believe in the benefits of a collective IS, such as EHRs, and those that are not. Such awareness can be of immense value to managers in developing strategies to introduce new technology and inspire change in workflow processes. Knowing that certain personality types will be inclined to have a negative attitude towards introducing a new IS might allow healthcare organizations to take the necessary steps to win them over. For instance, well-designed and well-structured training could significantly alter such individuals’ perspectives if the advantages are presented to appeal to the particular trainees. Incentive systems could also be usefully employed to target those individuals predisposed to resist the introduction of and/or struggle in learning to use technological innovations.

All factors contributing to the enhanced and continued use of new technology in organizational change-related management programs should be carefully considered. It is unlikely that one size can fit all concerning technological changes within an organization. Hence, the personality of individual users must be accounted for in every stage of the EHR implementation process.

### 6.3. Limitations and Future Research

As is always the case, the current study has limitations that should be considered when interpreting its findings. These limitations, however, also represent opportunities for future research.

First, this study focused on a sample population drawn only from Jordan’s public sector hospitals with fully implemented EHRs, and further research might also involve private hospitals. Similarly, the study limited its scope to one type of healthcare professional: nurses. Nonetheless, research into the behavior of physicians, pharmacists, physiotherapists, and laboratory technicians regarding EHR usage and the CI to use such would also be valuable.

Second, the survey method typically suffers from shortcomings like response bias and social desirability of responding to satisfy an author, misinterpretation and misunderstanding of the respondents concerning the questions, or false responses to questionnaire items. Indeed, the quantitative approach employed in this study to explore nurses’ CI to use EHRs and the role of personality therein is limited. To gain further insight into the subject, it would be helpful to consider a qualitative approach to gain further in-depth insights and provide superior support regarding the findings.

Third, this research employed a cross-sectional design, and it would thus be helpful to conduct a longitudinal study to validate the findings by testing the variables over time. Additionally, a cross-analysis of the results of the different levels of hospitals is highly recommended for future research.

Fourth, this study claims that the various theories integrated herein examine how individual-level factors such as personality are critical to the research on management ISs, especially in health informatics. Future research could build on this by considering, for example, how EHR adoption styles differ between early and late adopters. It would also be of value to ascertain how personality influences the UTAUT in a much more comprehensive framework.

Fifth, it would be interesting to examine the role of other personality factors concerning the UTAUT. While in psychology and other fields, FFM has driven theory development and inspired many empirical studies, management IS research has largely overlooked personality as a significant factor for a long time. Combining personality with other factors key to IS theory and examining their interrelation may significantly contribute to theoretical advances in this field.

Sixth, although this study examined nurses’ Continuance Intention (CI) to use EHRs through a combination of three conceptual frameworks: the Unified Theory of Acceptance and Use of Technology (UTAUT), the theory of expectation-confirmation, and the Five-Factor Model (FFM), it neglected many external key factors affecting users post-adoption such as satisfaction that future studies could investigate.

Finally, because of increasing concern about the nexus of human-computer interaction and IS studies, future examinations should be designed to investigate interventions and their impacts on IS continuance use using the framework provided in this study.

## Figures and Tables

**Table 1:** Hospitals Fully Implementing an Electronic Health Records System in Jordan with the Number of Nurses Employed.

Province	Hospital	Number of Nurses in the Hospital	Total Nurses in the Hospital	Minimum No, of Nurses to Be Collected from the Hospital
North	1	RN: 132	149	23
		Diploma: 17		
	2	RN: 126	137	21
		Diploma: 11		
	3	RN: 123	141	21
		Diploma: 18		
	4	RN: 90	110	17
		Diploma: 20		
	5	RN: 131	158	24
		Diploma: 27		
Central	6	RN: 444	528	79
		Diploma: 84		
	7	RN: 135	181	27
		Diploma: 46		
	8	RN: 363	472	71
		Diploma: 109		
South	9	RN: 186	226	34
		Diploma: 40		
	10	RN: 70	92	14
		Diploma: 22		
Total	10 hospitals	RN: 1800	2194	331
		Diploma: 394		

Note: RN and Diploma = 2194 [140].

**Table 2:** Demographic profile of respondents (n = 473).

Items	Category	N	%
Gender	Male	141	29.8
	Female Missing data	332 0	70.2 0
	Total	473	100
Age	20–30 years	208	44
	31–40 years	183	38.7
	41–50	75	15.9
	51–60	3	0.6
	Missing data	4	0.8
	Total	473	100
Education level	Diploma Degree	81	17.1
	Bachelor Degree	363	76.7
	Master Degree	24	5.1
	Ph.D. Degree	2	0.4
	Missing data	3	0.6
	Total	473	100
Profession Experience	1–5	128	27.1
	6–10	123	26
	11–15	100	21.1
	16–20	74	15.6
	21–25	38	8
	26–30	6	1.3
	31–35	1	0.2
	Missing data	3	0.6
	Total	473	100
Current Hospital Experience	1–5	220	46.5
	6–10	118	24.9
	11–15	60	12.7
	16–20	46	9.7
	21–25	19	4
	Missing data	10	2.1
	Total	473	100
Hospital	1	59	12.5
	2	23	4.9
	3	86	18.2
	4	36	7.6
	5	97	20.5
	6	29	6.1
	7	40	8.5
	8	23	4.9
	9	42	8.9
	10	38	8
	Missing data	0	0
	Total	473	100
Department	ICU	47	9.90
	CCU	24	5.10
	ER	52	10.1
	Dialysis	33	7.00
	Floor	223	47.1
	Operating room	94	19.9
	Missing data	0	0.00
	Total	473	100

**Table 3:** Loadings, Composite Reliability, and Average Variance Extracted.

Constructs	Measurement Items	Loadings	Cronbach’s Alpha	Composite Reliability (CR)	Average Variance Extracted (AVE)
Agreeableness	A1	0.810	0.734	0.830	0.552
	A10	0.762			
	A4	0.762			
	A7	0.626			
Conscientiousness	C12	0.856	0.885	0.831	0.567
	C7	0.864			
	C8	0.791			
	C9	0.406			
Continuous Intention	CI1	0.863	0.937	0.950	0.762
	CI2	0.856			
	CI3	0.837			
	CI4	0.899			
	CI5	0.907			
	CI6	0.873			
Extraversion	E11	0.775	0.719	0.827	0.545
	E4	0.648			
	E7	0.724			
	E8	0.798			
Effort expectancy	EE1	0.856	0.886	0.917	0.693
	EE2	0.897			
	EE3	0.885			
	EE4	0.637			
	EE5	0.859			
Facilitating Condition	FC1	0.823	0.875	0.909	0.666
	FC2	0.861			
	FC3	0.798			
	FC4	0.814			
	FC5	0.783			
Neuroticism	N11	0.765	0.798	0.856	0.544
	N12	0.647			
	N2	0.768			
	N6	0.809			
	N9	0.687			
Openness to Experience	O11	0.708	0.682	0.792	0.560
	O2	0.784			
	O3	0.753			
Performance Expectancy	PE1	0.900	0.883	0.927	0.810
	PE2	0.919			
	PE3	0.880			
Social Influence	SI1	0.637	0.886	0.910	0.562
	SI2	0.713			
	SI3	0.832			
	SI4	0.863			
	SI5	0.815			
	SI6	0.766			
	SI7	0.708			
	SI8	0.627			
Top Management Support	TMS1	0.737	0.879	0.902	0.537
	TMS2	0.777			
	TMS3	0.758			
	TMS4	0.707			
	TMS5	0.742			
	TMS6	0.791			
	TMS7	0.696			
	TMS8	0.644			

**Table 4:** Latent Variable Correlations and Square Roots of Average Variance Extracted.

Constructs	A	C	CI	E	EE	FC	N	O	PE	SI	TMS
A	0.743										
C	0.714	0.753									
CI	0.442	0.395	0.873								
E	0.670	0.695	0.396	0.739							
EE	0.479	0.391	0.712	0.440	0.832						
FC	0.400	0.302	0.518	0.315	0.558	0.816					
N	0.253	0.368	0.059	0.182	0.070	0.067	0.738				
O	0.678	0.650	0.417	0.679	0.470	0.357	0.182	0.749			
PE	0.393	0.332	0.766	0.350	0.635	0.480	0.046	0.388	0.900		
SI	0.408	0.325	0.544	0.328	0.541	0.520	−0.085	0.389	0.550	0.750	
TMS	0.363	0.206	0.439	0.298	0.486	0.652	−0.098	0.321	0.477	0.613	0.733

A = agreeableness, C = conscientiousness, E = extraversion, N = neuroticism, O = openness to experience, CI = continuous intention, EE = effort expectancy, FC = facilitating conditions, PE = performance expectancy, SI = social influence, and TMS = Top Management Support.

**Table 5:** Direct Relationship Hypotheses.

No.	Hypothesis	Beta	*p*-Value	*t* Statistic	Decision
H1	Effort Expectancy → Continuous Intention	0.314	0.000	5.284 ***	Supported
H2	Facilitating Conditions → Continuous Intention	0.091	0.025	2.246 **	Supported
H3	Performance Expectancy → Continuous Intention	0.491	0.000	10.425 ***	Supported
H4	Social Influence → Continuous Intention	0.075	0.080	1.756 *	Supported
H5	Top Management Support → Continuous Intention	−0.067	0.13	1.515	Not supported

Note: *t* values > 1.645 * (*p* < 0.05); *t* values > 1.96 ** (*p* < 0.02); *t* values > 2.33 *** (*p* < 0.01) 1-tailed test.

**Table 6:** Summary of Mediation Results.

					Bootstrapped Confidence Internal		
Hypothesis	Relationship	Indirect Effect (Beta)	SE	*t* Value	95% LL	95% UL	Decision
H6	A → PE → CI	0.104	0.035	2.958 ***	0.034	0.171	Supported
H7	C → PE → CI	0.018	0.037	0.487	−0.060	0.083	Not supported
H8	E → PE → CI	0.033	0.029	1.156	−0.021	0.095	Not supported
H9	N → PE → CI	−0.033	0.034	0.975	−0.093	0.040	Not supported
H10	O → PE → CI	0.092	0.036	2.575 ***	0.020	0.161	Supported

Note: *t* values > 1.96 * (*p* < 0.05); *t* values > 2.33 ** (*p* < 0.02); *t* values > 2.575 *** (*p* < 0.01) 2-tailed test. A = agreeableness, C = conscientiousness, E = extraversion, N = neuroticism, O = openness to experience, CI = continuous intention, SE = standard error, LL = lower limit, and UL = upper limit.

**Table 7:** Moderating Effects.

Hypotheses	Relationship	Beta	Standard Error	*t* Value	Decision
H11	EE → CI	−0.029	0.054	0.532	Not supported
H12	FCC → CI	−0.047	0.037	1.269	Not supported
H13	PEC → CI	−0.097	0.048	2.021 *	Supported
H14	SIC → CI	0.095	0.045	2.133 *	Supported

Note: *t* values > 1.96 * (*p* < 0.05); *t* values > 2.33 ** (*p* < 0.02); *t* values > 2.575 *** (*p* < 0.01) 2-tail test. EE = effort expectancy, FC = facilitating conditions, PE = performance expectancy, SI = social influence, C = conscientiousness, and CI = continuous intention.

**Table 8:** Results summary.

No.	Hypothesis	Beta	*t* Statistic	Decision
H1	EE → CI	0.314	5.284 ***	Supported
H2	FC → CI	0.091	2.246 **	Supported
H3	PE → CI	0.491	10.425 ***	Supported
H4	SI → CI	0.075	1.756 *	Supported
H5	TMS → CI	−0.067	1.515	Not supported
H6	A → PE → CI	0.104	2.958 ***	Supported
H7	C → PE → CI	0.018	0.487	Not supported
H8	E → PE → CI	0.033	1.156	Not supported
H9	N → PE → CI	−0.033	0.975	Not supported
H10	O → PE → CI	0.092	2.575 ***	Supported
H11	EE × C → CI	−0.029	0.532	Not supported
H12	FC × C → CI	−0.047	1.269	Not supported
H13	PE × C → CI	−0.097	2.021 **	Supported
H14	SI × C → CI	0.095	2.133 **	Supported

Note: *t* values > 1.96 * (*p* < 0.05); *t* values > 2.33 ** (*p* < 0.02); *t* values > 2.575 *** (*p* < 0.01) 2-tail test. EE = effort expectancy, FC = facilitating conditions, PE = performance expectancy, SI = social influence, A = agreeableness, C = conscientiousness, E = extraversion, N = neuroticism, O = openness to experience, CI = continuous intention.

## Data Availability

The data presented in this study are available on request from the corresponding author.
